# Modelling Cognitive Decline in the Hypertension in the Very Elderly Trial [HYVET] and Proposed Risk Tables for Population Use

**DOI:** 10.1371/journal.pone.0011775

**Published:** 2010-07-26

**Authors:** Ruth Peters, Nigel Beckett, Robert Beardmore, Rafael Peña-Miller, Kenneth Rockwood, Arnold Mitnitski, Shahrul Mt-Isa, Christopher Bulpitt

**Affiliations:** 1 Imperial Clinical Trials Unit, Imperial College London, London, United Kingdom; 2 Care of the Elderly, Imperial College London, London, United Kingdom; 3 Department of Mathematics, Imperial College London, London, United Kingdom; 4 Division of Geriatric Medicine, Dalhousie University, Halifax, Canada; 5 Department of Medicine, Dalhousie University, Halifax, Canada; National Institutes of Health, United States of America

## Abstract

**Introduction:**

Although, on average, cognition declines with age, cognition in older adults is a dynamic process. Hypertension is associated with greater decline in cognition with age, but whether treatment of hypertension affects this is uncertain. Here, we modelled dynamics of cognition in relation to the treatment of hypertension, to see if treatment effects might better be discerned by a model that included baseline measures of cognition and consequent mortality

**Methodology/Principal Findings:**

This is a secondary analysis of the Hypertension in the Very Elderly Trial (HYVET), a double blind, placebo controlled trial of indapamide, with or without perindopril, in people aged 80+ years at enrollment. Cognitive states were defined in relation to errors on the Mini-Mental State Examination, with more errors signifying worse cognition. Change in cognitive state was evaluated using a dynamic model of cognitive transition. In the model, the probabilities of transitions between cognitive states is represented by a Poisson distribution, with the Poisson mean dependent on the baseline cognitive state.

The dynamic model of cognitive transition was good (R^2^ = 0.74) both for those on placebo and (0.86) for those on active treatment. The probability of maintaining cognitive function, based on baseline function, was slightly higher in the actively treated group (e.g., for those with the fewest baseline errors, the chance of staying in that state was 63% for those on treatment, compared with 60% for those on placebo). Outcomes at two and four years could be predicted based on the initial state and treatment.

**Conclusions/Significance:**

A dynamic model of cognition that allows all outcomes (cognitive worsening, stability improvement or death) to be categorized simultaneously detected small but consistent differences between treatment and control groups (in favour of treatment) amongst very elderly people treated for hypertension. The model showed good fit, and suggests that most change in cognition in very elderly people is small, and depends on their baseline state and on treatment. Additional work is needed to understand whether this modelling approach is well suited to the valuation of small effects, especially in the face of mortality differences between treatment groups.

**Trial Registration:**

ClinicalTrials.gov NCT0012281

## Introduction

The ageing process is often associated with worsening health, however, the population is ageing and a means to identify those most at risk of cognitive decline or dementia, especially in the very elderly (aged 80 and over) category should be useful [1]. One technique which has been used for physical health is an index of frailty. The greater the number of deficits, (which can include symptoms, signs, diseases and disabilities) the greater the risk of developing further deficits and the possibility of incident deterioration in health or of death [2]–[4], [5]. Using a measure or index of frailty to describe transitions in health states, can include variation in health over time, including improvement [6]. Based on data from the Canadian Study of Health and Aging (CSHA) and that from the Gothenburg H70 study a mathematical model based on a Poisson distribution has been developed to account for the effect of number of deficits over time and their impact on subsequent mortality [6]. Following this, it has been suggested that a similar model could be applied to cognitive function and cognitive decline, such that the greater number of errors made on a cognitive test the more likely further decline at subsequent follow up. Using data from the CHSA baseline, five and 10 year visits a mathematical model of change in cognitive states, (the dynamic model of cognitive transition) was produced [7]. These cognitive states were based on the cognitive function scores from the Modified Mini-Mental State Examination (3MS) [8], [9]. The fit of the model was good (R^2^ = 0.96) and as this dynamic model of cognitive transitions accounted for both improvement and decline in cognitive function, it may be clinically relevant [7], [8]. Full details of the model used can be found in the methods section.

The CSHA sample was aged ≥65 years and less than 50 percent were hypertensive [10]. Whether this model would fit an older (>80 years) population, one that was hypertensive and whether the influence of anti-hypertensive treatment upon the probabilities of the model could be established remains to be seen. This is of interest as the fastest growing segment of society is aged 80 and over, both in developed and developing countries [11]. Due to the physiological effects of ageing, a large proportion of such older adults suffer from hypertension and there is evidence to suggest a link between high blood pressure and an increased risk of cognitive decline and dementia [12]. Further issues that combine to emphasize the importance of this health problem are the psychological, physical and financial burdens associated with caring for those with cognitive decline or dementia [11] and the recent results from the HYVET trial [13].

The Hypertension in the Very Elderly Trial [HYVET] was designed to examine the risks and benefits associated with the use of anti-hypertensive therapy in those aged 80 or more and has recently reported in favour of anti-hypertensive treatment reducing mortality, cardiovascular events, stroke and heart failure [13]. The HYVET trial also assessed cognitive function and did not find a statistically significant result using either interval censored or Cox proportional hazard survival analyses although the combination of the HYVET results with other similar trials in a meta-analysis did produce a summary ratio just significantly in favour of treatment [14]. If the dynamic model of cognitive transition fits with the HYVET data it could provide an opportunity to predict outcomes in very elderly hypertensive people. This high risk group would potentially allow modelling of the impact of antihypertensive treatment in a more sophisticated and potentially clinically applicable fashion. The HYVET data is taken from an older age group, a hypertensive group, predominantly female and with shorter follow up than the CSHA data set. The objective of this investigation was therefore to examine the applicability of this dynamic model of cognitive transitions and the impact of HYVET trial antihypertensive treatment upon cognitive functioning using methodology that may allow the evaluation of more subtle change than a traditional survival analysis. The model may also provide some means of predicting future cognitive health state based on baseline state and mortality risk. Two aims were pursued; 1: to evaluate the dynamic model of cognitive transitions with the HYVET data. 2: To use this modelling methodology to evaluate the impact of anti-hypertensive treatment in the HYVET population on cognitive functioning and to attempt to predict these effects.

## Methods

The Hypertension in the Very Elderly Trial was a double blind placebo controlled trial investigating the risks and benefits associated with the use of anti-hypertensive treatment in the very elderly. Full details of the protocol and results have already been published elsewhere [15], [16]. Briefly, in order to enter the trial participants had to be aged 80 or over at the time of randomisation, have a systolic blood pressure of > = 160mmHg and <200mmHg sitting and > = 140mmHg standing with a sitting diastolic pressure of <110mmHg. Participants could not enter HYVET if they required ongoing nursing care, were suffering from a condition that would severely limit their life or had received a clinical diagnosis of dementia. Treatment was with indapamide Slow Release[SR] 1.5mg, (or matching placebo) daily with the option of additional perindopril 2–4mg (or matching placebos) to reach a goal blood pressure of <150/80mmHg. Participants had their cognitive function assessed at baseline and annually thereafter with the Mini-Mental State Exam [MMSE]. Mortality data was also collected as part of the trial. The first description of the dynamic model of cognitive transition used the 3MS. The 3MS was split into categories to represent ranges of 3MS scores with clinically detectable differences in cognitive performance, i.e. to represent distinguishable cognitive health states [7], [8]. The model takes account of four factors, (1) the background expectation of accumulating additional errors, (2) the background and (3) increment in the probability of dying (based on the level of cognitive deficit) and (4) the increment in the expectation of incurring more or fewer errors, again based on the level of baseline cognitive deficits. In HYVET cognitive function data was collected using the MMSE which was similarly split into cognitive health states using ranges of the MMSE score (see below). The dynamic model of cognitive transitions was then applied to the HYVET data, using information from mortality and cognitive function assessments at baseline and after two and four year follow up, and change between these visits. Details of the model are below. Four year follow up was similar to that used in the CSHA and H-70 analyses and two year follow up was used because the participants were older, may show faster decline and because the mean follow up for the cognitive function aspect of HYVET trial was 2 years.

### Categorising cognitive health in HYVET

Cognitive health can be described in relation to an increasing number of cognitive deficits, represented by errors on a cognitive test. The MMSE in HYVET was categorised into those with 0–2 errors (cognitive health state 0), i.e. a score of 28–30, 3–5 errors (cognitive health state 1), a score of 25–27, 6–8 errors (cognitive health state 2), a score of 22–24, and so on. This is in line with the concept of deficit accumulation in relation to cognitive categories, as used with the 3MS data from the CSHA. Here, the categories are used in the model to measure the movement of participants between these cognitive health states. These categories of MMSE score were chosen to ensure clinical relevance and are similar to the frequently used cut-off values for MMSE score when used as a screening instrument for dementia [17]. The probability of changing from one cognitive state to another (in either direction, i.e. improved MMSE score resulting in a higher cognitive health state or a fall in MMSE score resulting in a lower cognitive health category) was then calculated for the HYVET data. Note that the model allows all clinically relevant outcomes (improvement, decline, stability, death) to be modelled simultaneously. The model was then applied to the HYVET database and further used to evaluate the effects of the active versus the placebo trial medication.

### Details of the model

We assume that the data collated can be described by a Markov process π consisting of the probabilities *p*_kn_ that a patient moves to class *k* from class *n* over a 24-month period, where the class ranges from 0 to 9. Each class represents a different *mental state* of the patient and is calculated from an MMSE score via an association
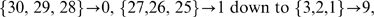
assuming a score of 0 cannot be achieved on an MMSE. Furthermore, for each mental state, *n*, there is an associated probability of death over a 24-month period for that state *p*_dn_, where the subscript d denotes death and not a numerical value.

As a result, the process π can be represented by an 11×11 *stochastic matrix* whose entries are the numbers *p*_kn_ and *p*_dn_:
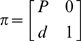
where the (*k*,*n*)-th entry in the 10×10 matrix *P* is *p_kn_* and d = (*p*_d0_, *p*_d1_,…, *p_d9_*). In this formulation death represents a state of each patient that once entered, cannot be escaped; such a state is called an absorbing state.

**Figure 1:**
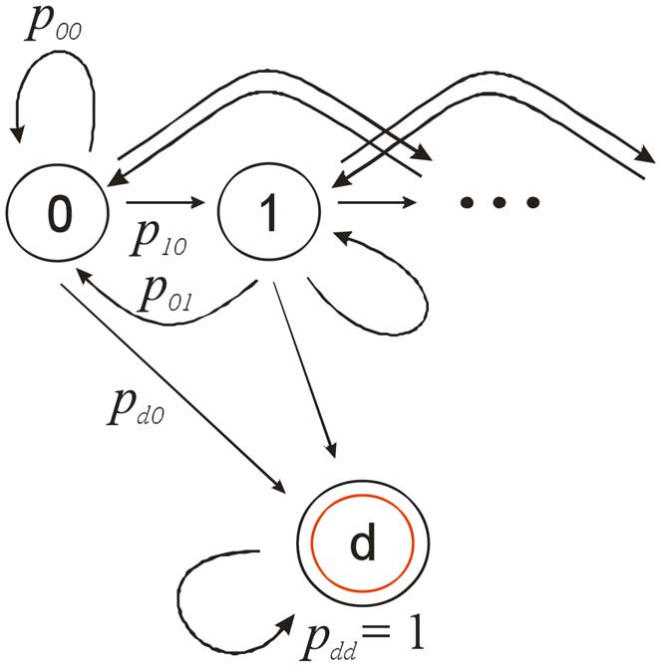
A diagram showing the basis of the model.

A schematic representation of three states that define the process π, is below illustrating some of the transitions between states 0, 1 and d, and their associated probabilities, state d is the absorbing state. The full model has eleven such states, see figure 1.

Following (Mitniski and Rockwood, 2008) [7] we assume a modified Poisson distribution for *p*_kn_ of the form
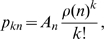
subject to P(death occurs | patients mental state at time zero is *n*) = *p*_dn_.

Conservation of probability then dictates
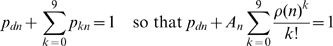
and the approximation
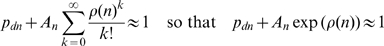
leads us to define 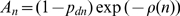. Finally therefore,
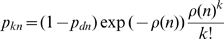
provides the values needed to define the process π.

A two-parameter exponential model [4] was used for the probability of death *p*_dn_, namely *p*_dn_ = αexp(β*n*) for parameters α and β that are first determined numerically from a least-squares fit to the death data of the 24-month survey for both active and placebo groups. In Mitniski, Bao and Rockwood, 2006 [2] the 2-parameter model
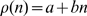
was proposed and so we also adopt this form for ρ(*n*) which renders *p_kn_* (and therefore **π** also) a function of the parameters *a* and *b*.

We then determine the parameter pair (*a*, *b*) so that the least squares residual
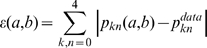
is minimised, noting that the summation terminates at state 4 and not 9 because MMSE data is only available for those states. The term *p_kn_* ^data^ denotes the frequency of patients surveyed and found to be in mental state *k* at a time 24 months after the initial MMSE test at which the patient was in state *n*.

Due to the approximations made, this minimisation procedure does not necessarily result in a stochastic matrix π and there are different remedies to this; we chose simply to scale each column of π to sum to unity in order to enforce stochasticity.

The software packages used were Matlab and SAS v9.1.

The funders had no role in study design, data collection and analysis, decision to publish, or preparation of the manuscript.

## Results

The HYVET trial randomised 3845 participants of whom 3336 provided longitudinal MMSE data. There were 739 participants available for analysis in the active group at two years and 699 in the placebo group with the majority in the cognitive health states representing better cognitive functioning.

### Model

**Figure 2:**
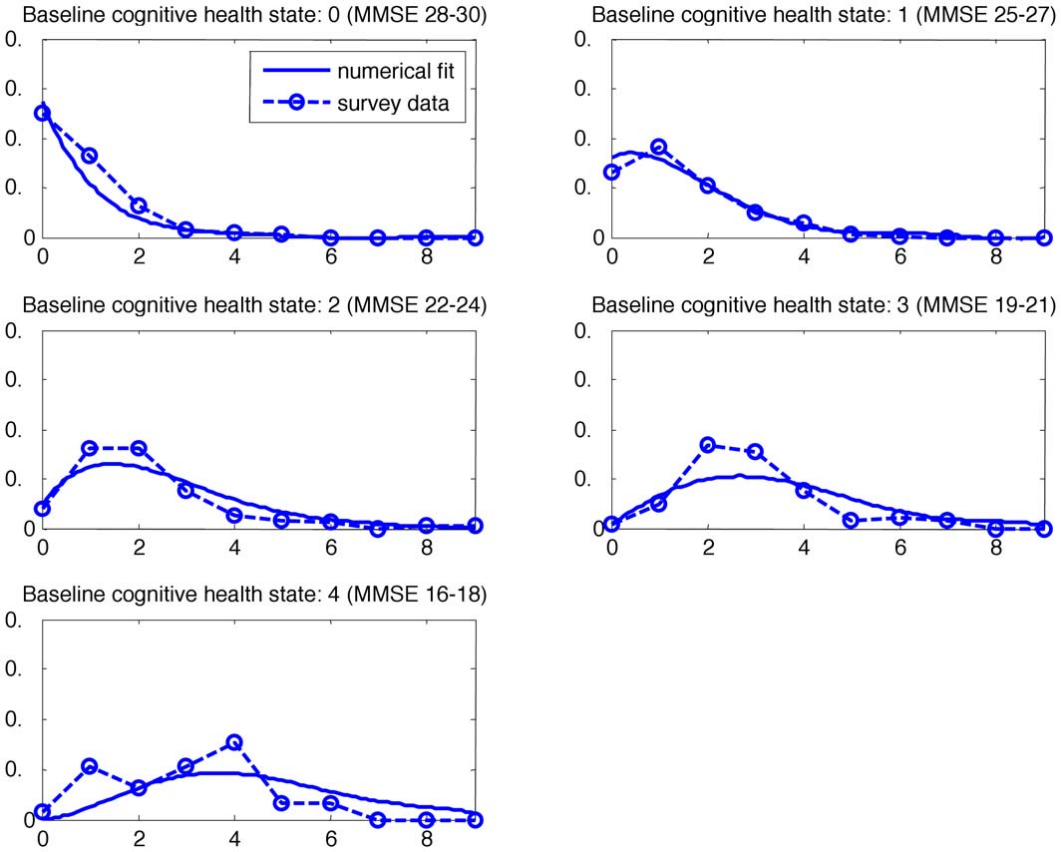
A graph showing change in health state (placebo group) at two years, by baseline health state.

The dynamic model of cognitive transitions was applied to the HYVET data and used to create a plot showing the change in health state, by baseline health state and per treatment group. The results can be seen in figures 2 (placebo group) and 3 (active group). The graphs show both the surveyed (i.e. collected data) and that predicted by the model (i.e. a fitted distribution). In each figure there are five graphs, the first graph represents those participants who were in cognitive health state 0 (MMSE 28–30) at the trial baseline. The x axis shows the cognitive health state that they had attained after two year follow up and the probability of falling into the later cognitive health state is shown on the y axis. Thus it can be seen that the majority of those participants who started the trial in cognitive health state 0, were cognitively intact and remained so after two years. The data from the trial participants is indicated by circles on the graph. The second graph presents the same information for those who entered the trial in cognitive health state 1 and so on for both the placebo and actively treated groups. Participants could not enter the HYVET trial if they had a clinical diagnosis of dementia and this meant that the full range of MMSE scores was not present at trial baseline.

**Figure 3:**
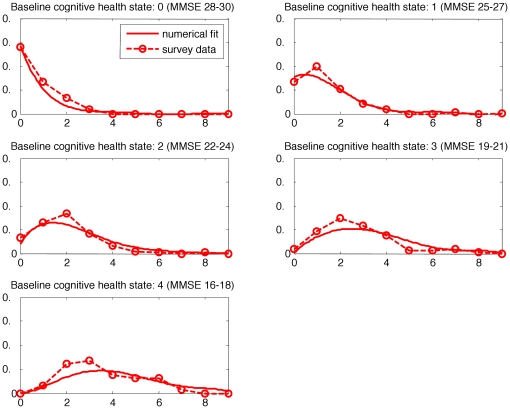
A graph showing change in cognitive health state (active group) at two years, by baseline health state.

In both treatment groups it can be seen that the higher the baseline cognitive health state the higher the likelihood of remaining in that cognitive health state, although it should be remembered that the maximum score on the MMSE is 30 and that ceiling effects are likely to apply. The model showed a relatively good fit to the HYVET data with the R^2^ for the two year follow up data at R^2^ = 0.74 for the placebo group and R^2^ = 0.86 for the active group. In order to test the model further it was extrapolated to predict four year follow up data which was then compared back to the collected, i.e. real data from the trial. Although the numbers for analysis in the trial at four year follow up (342) were less than at two years, this still resulted in R^2^ values of R^2^ = 0.78 for the placebo group and R^2^ = 0.72 for the active group.

### Predicting cognitive health state after two year follow up

The model was then used to predict the probability of moving from one health state to the next in numerical form thus allowing the creation of a ‘risk table’ such that future health/cognitive state can be predicted based on current state and taking account of antihypertensive treatment. Tables 1 and 2 provide these probabilities. Each treatment group is represented by one table where the columns represent the cognitive health state at baseline and the rows the cognitive health state attained at two years. For example, the probability of remaining in cognitive health state 0 at two years and in the placebo group (table 1) is 0.60. The tables show that, according to the model as based on the HYVET data, the probability of maintaining cognitive function based on baseline function may be slightly higher in the actively treated group. The actively treated group maintained a very slightly higher probability of remaining in their baseline cognitive health states. Although the figures in the table are derived from the model the patterns were very similar when examining the raw data, (for example the probability of remaining in cognitive health state 0 was 0.559 in the actively treated group versus 0.497 in the placebo group). Numbers were small however and so further analysis using bootstrapping techniques and the adjusted bootstrap percentile method was carried out. This resulted in means and 95% confidence intervals for change in MMSE score of (a fall of) 1.91 (1.63–2.23) for the active group, compared to 2.03 (1.72–2.38) for the placebo group.

**Table 1:** Change in MMSE over two years - placebo group.

	Probability of moving from (baseline) MMSE score…					
	Cognitive health state	MMSE 28–30 0	MMSE 25–27 1	MMSE 22–24 2	MMSE 19–21 3	MMSE 16–18 4
**to MMSE score at two years of…**	MMSE 28–30 **0**	0.60	0.29	0.09	0.02	0.00
	MMSE 25–27 **1**	0.23	0.30	0.23	0.12	0.05
	MMSE 22–24 **2**	0.09	0.19	0.23	0.20	0.12
	MMSE 19–21 **3**	0.03	0.10	0.17	0.20	0.17
	MMSE 16–18 **4**	0.01	0.05	0.11	0.16	0.18
	MMSE 13–15 **5**	0.01	0.02	0.06	0.11	0.15
	MMSE<13 **>5**	0.00	0.02	0.06	0.14	0.26
	**death**	0.02	0.03	0.04	0.05	0.06
**Mortality**		0.02	0.03	0.04	0.05	0.06
**Cognitive decline**		0.38	0.38	0.41	0.42	0.41
**Cognitive Improvement/Stability**		***0.60***	***0.59***	***0.55***	***0.53***	***0.53***

**Table 2:** Change in MMSE over two years - active group.

	Probability of moving from (baseline) MMSE score…					
	Cognitive health state	MMSE 28–30 0	MMSE 25–27 1	MMSE 22–24 2	MMSE 19–21 3	MMSE 16–18 4
**to MMSE score at two years of…**	MMSE 28–30 **0**	0.63	0.30	0.08	0.01	0.00
	MMSE 25–27 **1**	0.22	0.32	0.25	0.13	0.05
	MMSE 22–24 **2**	0.08	0.19	0.25	0.22	0.15
	MMSE 19–21 **3**	0.03	0.09	0.17	0.21	0.20
	MMSE 16–18 **4**	0.01	0.04	0.10	0.16	0.19
	MMSE 13–15 **5**	0.00	0.02	0.05	0.10	0.15
	MMSE<13 **>5**	0.00	0.01	0.05	0.12	0.23
	**death**	0.02	0.02	0.04	0.03	0.02
**Mortality**		0.02	0.02	0.04	0.03	0.02
**Cognitive decline**		0.35	0.36	0.37	0.38	0.38
**Cognitive Improvement/Stability**		***0.63***	***0.62***	***0.58***	***0.58***	***0.59***

**Figure 4:**
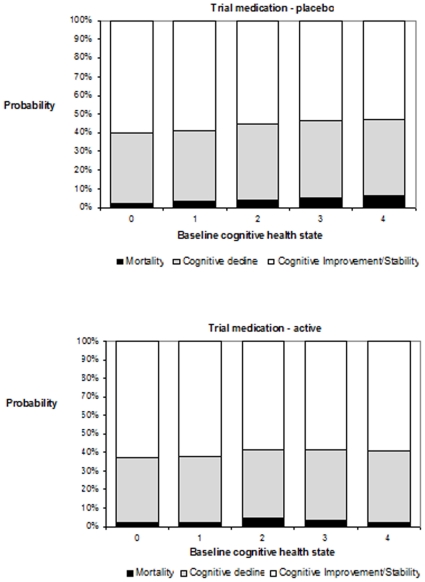
Probability of change over two years by baseline health state.

Figure 4 presents the same information in the form of ‘bars’ of probability with the actively treated group demonstrating a greater probability of participants remaining in the upper, cognitively stable section.

Details of the modelling of the four year data and the corresponding graphs are available in Appendix S1 and Figures S1 and S2.

### Mortality

When mortality was examined, the risk of mortality in the placebo group was greater in those with greater cognitive deficits. This pattern was not clearly seen in the active group although it must be remembered that active treatment reduced mortality in the trial overall.

## Discussion

The HYVET data on cognitive health state change and based on cognitive health state fits with the recently proposed dynamic model of cognitive transitions. The innovative aspect of our analysis is the incorporation of mortality in the assessment and we consider that this will be welcomed by researchers and therapists alike. Furthermore, the application of the model to the HYVET data suggests that there is a possibility that anti-hypertensive treatment may moderate cognitive decline, although this needs to be confirmed by additional studies. Even so, these analyses suggest that people treated with anti-hypertensives may maintain their cognitive health state for longer, something that was not shown statistically here but which clearly merits further investigation. If, as is implied by our findings, anti-hypertensive treatment slows or prevents further decline, it may be useful in terms of allowing those with early cognitive decline to function independently for longer or act as an incentive for patients to adhere with treatment regimens. The tables of probabilities allow some element of prediction and may be useful for health care professionals planning future patient reviews, resources, and initiating currently available or even future treatments. Again, this utility needs to be demonstrated in further studies. It is argued that the table that should receive the greatest focus is that from the actively treated group since the HYVET trial clearly showed benefit from treatment in elderly hypertensives for mortality, stroke and heart failure and such treatment may be expected to be given in the future. Further exploration of other factors that may impact upon cognitive function would be a useful addition to such tables as would information relating to change in profile of risk factors with increasing age, such as cholesterol level, risk behaviours such as smoking etc.

There are several benefits associated with the use of this model. First, it allows representation of change in all directions (improvement, decline and mortality) simultaneously, as competing events. Second, it allows for graduated changes in cognition (both declines and improvements) to be assessed. Third, it is parsimonious; it is based on Poisson distribution. Fourth, it allows precise parameters estimation. The technique can be used by using Poisson regression models that are readily available in many statistical software packages as a part of Generalized Linear Model programming. Fifth, it is easily interpretable. The intercepts of the Poisson mean and of the probability of death characterize transition of the people who had the best cognitive performance at baseline and therefore can be used in studying the factors influencing such performance to better understanding the initial stages of impairment. Despite this, however, our analyses must be interpreted with caution. As the method is new, it requires additional use to understand its full range of advantages and limitations.

In addition, we are limited by having the MMSE as the global cognitive measure. Whilst a good pragmatic choice, it is not an ideal assessment of cognitive functioning and suffers from ceiling effects, even in very elderly people [9], [17]. It may also be necessary to follow participants for longer than two years in order to fully evaluate the impact of any treatment. Because HYVET was stopped early this was not possible in this cohort. It is argued that this area merits a study with the focus upon change in cognitive function as an outcome and the impact of anti-hypertensive treatment and other risk factors and interventions examined. Further research and collaboration is also required in order to expand the ‘risk tables’ presented here to incorporate other key factors in cognitive ageing and allow potential targeted interventions as and when further treatments to ameliorate or prevent cognitive decline become available.

### Conclusions

Using a model which takes into account risk of mortality and of increase as well as decrease in cognitive performance data from the Hypertension in the Very Elderly [HYVET] Trial suggests that antihypertensive treatment may reduce risk of cognitive decline. Caution must be applied as the differences between the active and placebo groups were not large or significantly different and mortality was reduced with active treatment, however, it is argued that this highlights the need for further and more sophisticated studies. Such future work should include exploration of risk factors that could allow the development of risk tables for cognitive decline.

## Supporting Information

Appendix S1 Appendix detailing further supporting information.
(0.04 MB DOC)

Figure S1 A graphical representation of the model and the data for the placebo group at 48 months.
(0.03 MB TIF)

Figure S2 A graphical representation of the model and the data for the active group at 48 months.
(0.03 MB TIF)
