# Supplementary material for: Modelling Cognitive Decline in the Hypertension in the Very Elderly Trial [HYVET] and Proposed Risk Tables for Population Use
Source: PLoS One. 2010 Jul 26;5(7):e11775. doi: 10.1371/journal.pone.0011775 (PMC2909901; doi:10.1371/journal.pone.0011775)
Supplement: Appendix S1 — Appendix detailing further supporting information. (0.04 MB DOC) [file pone.0011775.s001.doc]

**Appendix**

For group 1 (placebo) we obtained the parameter set {α, β, *a*, *b*} = {0.0188, 0.2460, 0.58345, 0.94046} to provide an estimated Markov process of cognitive function decline *without* treatment, **π**_1_, where R^2^ = 0.74 and ε(*a,b*) = 4.1.

For group 2 (active treatment) we obtained the parameter set {α, β, *a*, *b*} = {0.0258, 0.0336, 0.53126, 1.0327} to provide an estimated Markov process of cognitive function decline *with* treatment, **π**_2_, where R^2^ = 0.86 and ε(*a,b*) = 3.46.

We compared the results obtained by applying both matrices **π**_1_ and **π**_2_ to the 24-month survey data for each group, 1 and 2 respectively, to predict mental state distribution at 48 months. Comparing these predictions against surveyed MMSE data at 48 months provides R^2^ values 0.78 for group 1 data using **π**_1_, R^2^ = 0.72 for group 2 data using **π**_2_. The predicted distributions and survey data are shown in Figures 5 and 6 for several baseline states.
