# Supplementary figures and images for: Modelling Cognitive Decline in the Hypertension in the Very Elderly Trial [HYVET] and Proposed Risk Tables for Population Use
Source: PLoS One. 2010 Jul 26;5(7):e11775. doi: 10.1371/journal.pone.0011775 (PMC2909901; doi:10.1371/journal.pone.0011775)

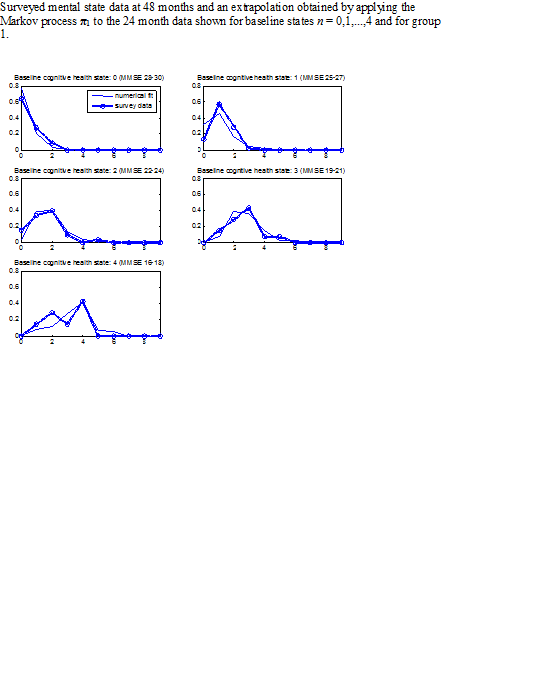

Supplement: Figure S1 — A graphical representation of the model and the data for the placebo group at 48 months. (0.03 MB TIF) [file pone.0011775.s002.tif]

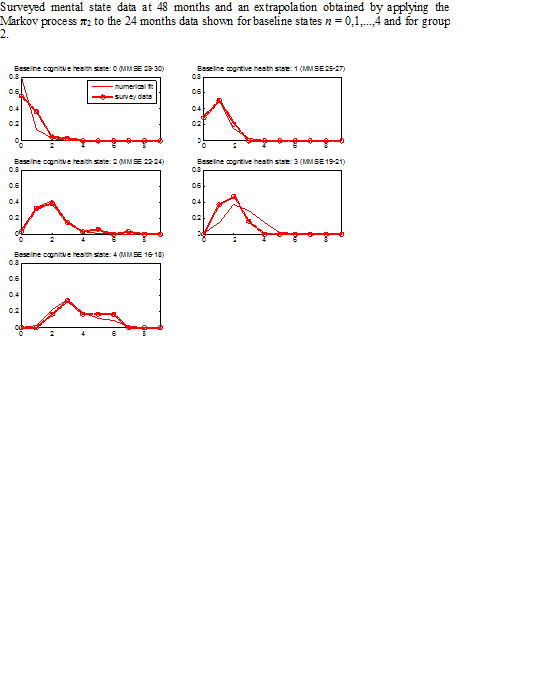

Supplement: Figure S2 — A graphical representation of the model and the data for the active group at 48 months. (0.03 MB TIF) [file pone.0011775.s003.tif]
